# Metastatic Melanoma Prognosis Prediction Using a TC Radiomic-Based Machine Learning Model: A Preliminary Study

**DOI:** 10.3390/cancers17142304

**Published:** 2025-07-10

**Authors:** Antonino Guerrisi, Maria Teresa Maccallini, Italia Falcone, Alessandro Valenti, Ludovica Miseo, Sara Ungania, Vincenzo Dolcetti, Fabio Valenti, Marianna Cerro, Flora Desiderio, Fabio Calabrò, Virginia Ferraresi, Michelangelo Russillo

**Affiliations:** 1 Radiology and Diagnostic Imaging Unit, Department of Clinical and Dermatological Research, San Gallicano Dermatological Institute IRCCS, 00144 Rome, Italy; antonino.guerrisi@ifo.it (A.G.); ludovica.miseo@ifo.it (L.M.); flora.desiderio@ifo.it (F.D.); 2 Departement of Clinical and Molecular Medicine, Università La Sapienza di Roma, 00185 Rome, Italy; mariateresa.maccallini@ifo.it (M.T.M.); marianna.cerro@ifo.it (M.C.); 3 SAFU, Department of Research, Advanced Diagnostics and Technological Innovation, IRCCS-Regina Elena National Cancer Institute, 00144 Rome, Italy; 4 Medical Physics and Expert Systems Laboratory, Department of Research and Advanced Technologies, IRCCS-Regina Elena Institute, 00144 Rome, Italy; sara.ungania@ifo.it; 5 Department of Radiological, Anatomo-Pathological Sciences, “Sapienza” University of Rome, Viale Regina Elena 324, 00161 Rome, Italy; vincenzo.dolcetti@uniroma1.it; 6 UOC Oncological Translational Research, IRCCS-Regina Elena National Cancer Institute, 00144 Rome, Italy; fabio.valenti@ifo.it; 7 Medical Oncology 1, IRCCS-Regina Elena National Cancer Institute, 00144 Rome, Italy; fabio.calabro@ifo.it; 8 Sarcomas and Rare Tumors Departmental Unit, IRCCS-Regina Elena National Cancer Institute, 00144 Rome, Italy; virginia.ferraresi@ifo.it (V.F.); michelangelo.russillo@ifo.it (M.R.)

**Keywords:** metastatic melanoma, radiomics features, artificial intelligence, prognosis, patients stratification

## Abstract

**Background/Objective**: The approach to the clinical management of metastatic melanoma patients is undergoing a significant transformation. The availability of a large amount of data from medical images has made Artificial Intelligence (AI) applications an innovative and cutting-edge solution that could revolutionize the surveillance and management of these patients. In this study, we develop and validate a machine-learning model based on radiomic data extracted from a computed tomography (CT) analysis of patients with metastatic melanoma (MM). This approach was designed to accurately predict prognosis and identify the potential key factors associated with prognosis. **Methods**: To achieve this goal, we used radiomic pipelines to extract the quantitative features related to lesion texture, morphology, and intensity from high-quality CT images. We retrospectively collected a cohort of 58 patients with metastatic melanoma, from which a total of 60 CT series were used for model training, and 70 independent CT series were employed for external testing. Model performance was evaluated using metrics such as sensitivity, specificity, and AUC (area under the curve), demonstrating particularly favorable results compared to traditional methods. **Results**: The model used in this study presented a ROC-AUC curve of 82% in the internal test and, in combination with AI, presented a good predictive ability regarding lesion outcome. **Conclusions**: Although the cohort size was limited and the data were collected retrospectively from a single institution, the findings provide a promising basis for further validation in larger and more diverse patient populations. This approach could directly support clinical decision-making by providing accurate and personalized prognostic information.

## Simple Summary

Although much progress has been made, melanoma still remains a disease with an often poor prognosis. So, identifying new imaging markers that are able to give prognosis predictions would help in the management of patients and would avoid unnecessary (and often harmful) treatments. With the aim of providing early and accurate prediction of clinical outcome, in this preliminary study, we developed a machine learning model based on radiomic features extracted from CT images of patients with metastatic melanoma. Through the use of radiomics, we have the ability to reveal aspects of the tumor not visible to the human eye. Integrated with artificial intelligence, it improves predictive ability and promotes personalized treatment choices. Although this is a pilot study, the results offer a promising basis for future multicenter validations.

## 1. Introduction

Metastatic melanoma (MM) is characterized by significant intrinsic heterogeneity in secondary lesions, presenting unique challenges for patient management and treatment selection. Within a single melanoma tumor, distinct populations of cancer cells often display diverse genetic, molecular, and phenotypic characteristics [1]. This heterogeneity enables some cells to resist treatment, evade immune responses, and adapt to changing environments. Intra-tumoral heterogeneity is driven by genetic mutations, epigenetic alterations, and interactions with the tumor microenvironment, collectively contributing to melanoma’s resilience against therapy and complicating complete eradication [2]. Inter-tumoral heterogeneity further complicates disease management because different lesions may exhibit varying mutations, growth rates, and treatment responses [3]. Factors such as the tumor’s site of origin influence its progression and behavior [4]. This diversity necessitates moving away from one-size-fits-all approaches, as heterogeneity contributes to poor prognosis prediction and therapeutic resistance, particularly to targeted therapies and immunotherapies [5]. State-of-the-art advances in these treatment modalities are improving survival rates and, in some cases, have achieved durable responses [6]. However, individualized treatment planning remains essential due to the variability in therapeutic efficacy and its associated adverse effects [7]. MM management also requires frequent follow-ups to optimize treatment selection, imposing a significant burden on national healthcare systems. Striking a balance between clinical efficacy, patient safety, and economic sustainability is, therefore, critical [8]. Currently, the main criteria to choose therapeutic options for MM patients at high risk of poor outcomes include the presence of BRAF mutations, overall clinical condition, and the number of liver metastases, particularly if this number greater than three [9,10]. Therefore, the lack of widely accepted prognostic criteria is an unsolved problem in MM patient management, and the development of reliable prognostic indicators is crucial in optimizing treatment strategies and improving outcomes in MM treatment. Radiomics, applied to pre-treatment CT imaging, represents a promising approach to address this gap in prognostic stratification [11]. Recent studies have confirmed the value of radiomic signatures extracted from CT scans in identifying prognostic patterns in metastatic melanoma. For instance, Dercle et al. (2022) developed a radiomic model capable of predicting 6-month overall survival (OS) in advanced melanoma patients treated with anti-PD-1 therapy, achieving an AUC of 0.92 and outperforming conventional RECIST-based assessments [12]. Similarly, Brendlin et al. (2021) demonstrated that CT dual-energy radiomics could significantly improve the prediction of immunotherapy response in stage IV melanoma patients, particularly when assessing lesion-specific heterogeneity [13]. Nevertheless, evidence for this is not fully concordant. While Wang et al. (2020) showed promising results using a delta-radiomics model to identify early responders to immunotherapy, larger multicenter studies, such as those by Ter Maat et al. (2023) and Peisen et al. (2023), found that radiomic features alone or in combination with clinical data did not significantly improve prognostic prediction over traditional clinical models [14,15,16]. These discrepancies underline the need for the further validation and refinement of radiomic models, especially when used in clinical decision-making for treatment planning and outcome prediction in MM treatment. Indeed, radiomics is proven to be able to capture intra-tumoral heterogeneity that is driven by genetic mutations, epigenetic alterations, and interactions with the tumor microenvironment, both in vivo and non-invasively. Thus, by extracting quantitative data from pre-therapy computed tomography (CT) scans, radiomics enables the identification of lesion-specific characteristics that may correlate with favorable or unfavorable prognosis at an early stage in patient management. This technique can offer an objective and reproducible tool for risk assessment, complementing traditional clinical and molecular criteria. Moreover, integrating radiomic analysis into MM management has the potential to enhance treatment personalization, enabling clinicians to tailor interventions based on a patient’s unique lesion profile and, ultimately, improving clinical outcomes [17].

The aim of this study is to assess the potential of a radiomic-based model for the improvement of MM management. For this purpose, a radiomic-based machine-learning model was trained and tested on pre-therapy CT images through the study of metastatic lesions to classify prognosis in IV stage melanoma subjects and to provide a more comprehensive prognostic evaluation.

## 2. Materials and Methods

### 2.1. Study Population

In this retrospective study, a first dataset of 60 CT series of metastatic lesions were collected from 38 MM patients (Table 1), 32 lesions associated with “favorable prognosis” (FP) and 28 to “unfavorable prognosis” (UP), in which prognosis was assessed with progression free survival (PFS) </> treatment median PFS. This dataset was used as the training and internal testing datasets of the radiomics-based models.

A second dataset of 70 CT series of metastatic lesions were collected from an independent cohort of 20 MM patients (Table 1); this second dataset was used to externally test the trained radiomics-based models. All patients included in this study (in the internal and external testing) were treated with targeted therapy and immuno-checkpoint inhibitors (ICIs), and the median PFS of each therapy was related to the main randomized clinical trials [17,18,19,20,21,22,23].

### 2.2. Radiomics Analysis and Model Construction

**Figure 1:**
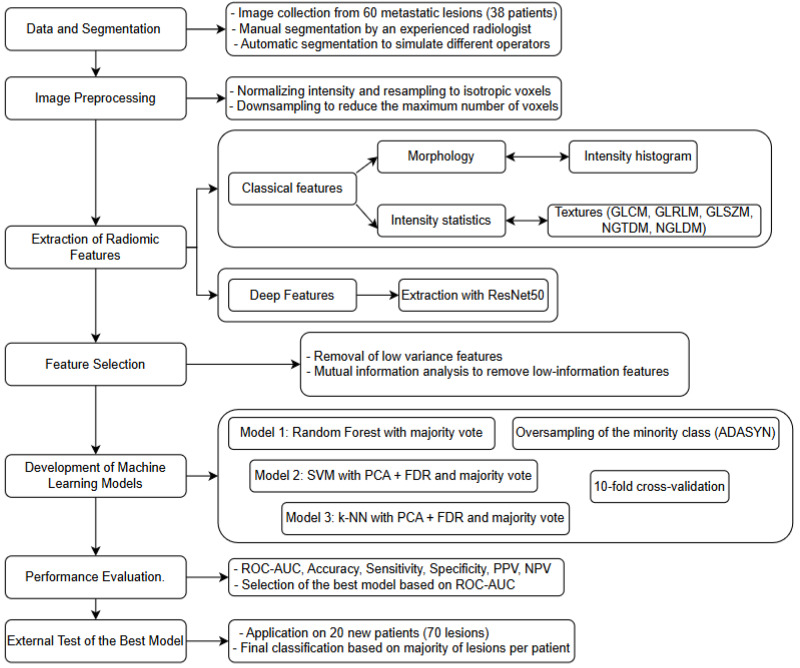
Analysis workflow.

In order to test the three machine-learning models, a set of segmented images was used for training and cross-validation by the software, resulting in a fully automated, consistent workflow that complies with IBSI standards. Radiomics is fundamentally based on the extraction of imaging features imperceptible to the naked eye that may serve as potential indicators of heterogeneity between FP and UP classes. Specifically, the workflow was articulated in the following points summarized in Figure 1.

**Figure 2:**
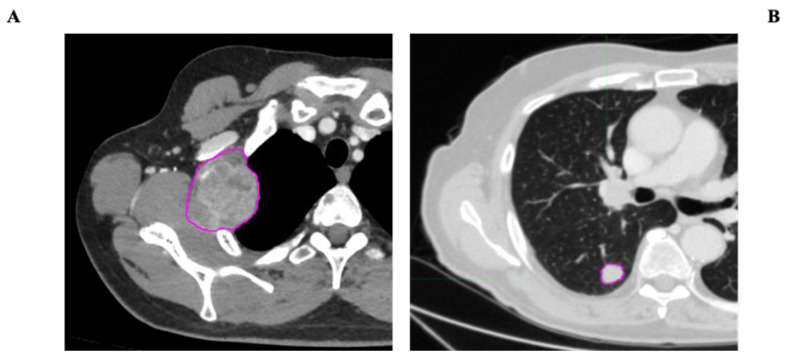
Examples of metastatic lesion segmentation, with pink contours delineating ROIs (regions of interest). (**A**) Heterogeneous-density solid lesion of the right lateral thoracic wall. (**B**) Nodular lesion of the right lung parenchyma.

1. VOI segmentation: Segmentation of the Volume of Interest (VOI) was performed manually on a slice-by-slice basis in consensus by two expert radiologists in CT imaging (15 and 10 years of experience) using the Trace4Research segmentation tool (named Manual segmentation by an experienced radiologist) (Figure 2).

The software provides additional automatic segmentations from random manipulation of the human expert segmentation in order to mimic different physicians in this task and to derive other segmentations.

2. Pre-processing of image intensities: To account for the potential variability introduced by the heterogeneous acquisition parameters, the intensity values within the segmented Volume of Interest (VOI) were pre-processed by resampling all CT series of each patient at an isotropic voxel spacing of 1 × 1 × 1 mm^3^, ensuring spatial consistency between datasets. Additionally, a voxel count limitation was applied to standardize computational load and reduce potential bias due to lesion volume differences: texture features were extracted from a maximum of 10 million voxels per VOI, while shape and first-order features were calculated on VOIs sampled up to a maximum of 1 million voxels. Voxel intensities within the segmented VOIs were quantized using fixed-width bins (64 bins for traditional radiomic features; 256 bins for deep features), enabling consistent intensity-based feature calculations in all cases. All preprocessing steps were carried out the Trace4Research platform, following a workflow aligned with Image Biomarker Standardization Initiative (IBSI) recommendations. This standard approach, commonly adopted for this type of data, helped to harmonize the feature space across cases, while also compensating for significant lesion size variations.
3. Radiomic feature extraction: Radiomic features were extracted from the segmented VOI across multiple feature families:
  - Morphology.
  - Intensity-based statistics.
  - Intensity histogram (computed after discretizing the VOI intensities into 64 fixed bins).
  - Texture features, including the following: Gray-Level Co-occurrence Matrix (GLCM), Gray-Level Run Length Matrix (GLRLM), Gray-Level Size Zone Matrix (GLSZM), Neighborhood Gray-Tone Difference Matrix (NGTDM), and Neighboring Gray-Level Dependence Matrix (NGLDM).
  - Deep features: A set of 2048 deep features was extracted using the convolutional layers of a pre-trained ResNet50 model. The input images were resampled to dimensions of 224 × 224 × 16 voxels and discretized into 256 fixed intensity bins (these features are not included in the IBSI guidelines).

Steps 2 and 3 were performed using the Trace4Research Radiomics tool, and the extracted features were reported following IBSI standards.

4. Feature selection: Low-variance features (variance < 0.1) were removed, followed by a mutual-information analysis to exclude features with low association with the class label (mutual information < 0.39). This selection pipeline ensured the inclusion of features with significant discriminant power while minimizing overfitting. All steps were performed within the Trace4Research platform and, where applicable, were aligned with the IBSI standard-compliant methodology.
5. Machine learning classification models: Three machine-learning classifiers were developed, validated, and tested for the binary classification task (FP vs. UP), using prognosis as the reference standard. A nested 10-fold cross-validation strategy was applied for all models:
  - Model 1: Four ensembles of random forest classifiers, combined with the Gini index and a majority-vote rule.
  - Model 2: Four ensembles of support vector machines (SVMs), combined with principal component analysis (PCA) and the Fisher Discriminant Ratio (FDR), using a majority-vote rule.
  - Model 3: Four ensembles of k-nearest neighbor (k-NN) classifiers, also combined with PCA, FDR, and a majority-vote rule. To address potential bias due to class imbalance, the Adaptive Synthetic Sampling technique (ADASYN) was applied to oversample the minority (poor prognosis) class, ensuring a more balanced representation during model training. Model performance was evaluated using several metrics, including overall accuracy, area under the ROC curve (ROC-AUC), specificity, sensitivity, Negative Predictive Value (NPV) and Positive Predictive Value (PPV). For each of these metrics, 95% confidence intervals were calculated to provide a reliable estimate of their variability. Among all the models analyzed, the best performing binary classifier was selected based on the highest ROC-AUC value. The best-performing model, according to the ROC-AUC value of the internal testing, was then externally tested on the 20 MM patients of the external testing dataset (for a total of 70 lesions) using the most significant predictors. The classification of each patient’s prognosis was obtained as the one most frequently assigned by the classifier to the metastatic lesions of the same patient.

### 2.3. Statistical Analysis

To describe in detail the distribution of the most significant radiomic features in the “FP” and “UP” classes, median values accompanied by 95% confidence intervals were calculated. Violin and box plot graphs were made to provide an immediate visual understanding of the variability and distribution of the data. A univariate nonparametric test, the Wilcoxon rank-sum test (also known as the Mann–Whitney U test), was applied to assess the discriminating ability of each radiomic predictor in distinguishing between the “FP” and “UP” classes; moreover, these data do not follow a normal distribution, which, this test indicated, ensured robustness in the analysis. To control the risk of false positives, a correction of *p*-values was adopted using the Bonferroni–Holm method, a less conservative procedure than the classic Bonferroni, that increases statistical power while maintaining control over type I errors. Significance levels were set at 0.05 (*) and 0.005 (**), thus providing two thresholds for identifying statistically significant and highly significant results, respectively. This procedure ensured the reliability of the results obtained, allowing for the precise identification of the radiomic features most useful for prognostic classification between the two classes of interest. Statistical analysis was performed using the tools integrated in the Trace4Research platform, which allowed for comprehensive and automated data management.

## 3. Results

The performance metrics of the three developed models are shown in Table 2 for the training, validation and internal testing processes.

**Figure 3:**
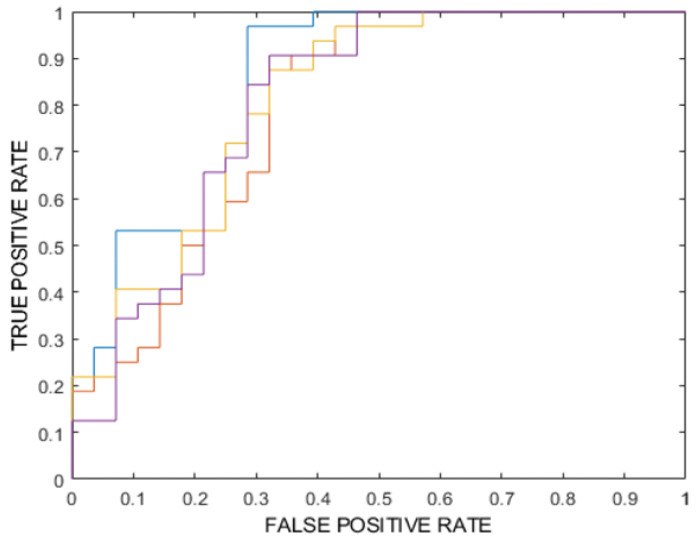
ROC curves for the best-performing model (random forest ensemble) obtained from internal testing. Each colored line represents the ROC curve of a different classifier within the ensemble or from different validation folds, showing variability in performance across internal test subsets. The curves illustrate the trade-off between true positive rate (y-axis) and false positive rate (x-axis) for various classification thresholds.

Internal testing performance is shown for both the mean vote among the classifiers of each model and the majority vote. The best model according to internal testing (composed of four ensembles of random forest) showed a ROC-AUC (%) of 82 (majority vote), 81.6 ** (mean) [77.9–85.4], accuracy (%) of 77, 75.4 ** [74.1–76.7], sensitivity (%) of 84, 80.5 ** [78–83], specificity (%) of 68, 69.6 ** [66.4–72.9], Positive Predictive Value (PPV) (%) of 75, 75.2 ** [73.5–76.9] and Negative Predictive Value (NPV) (%) of 79, 75.7 ** [73.8–77.7] (the significance levels are as follows: * *p*-value < 0.05, ** *p*-value < 0.005). ROC-AUC is reported in Figure 3.

**Figure 4:**
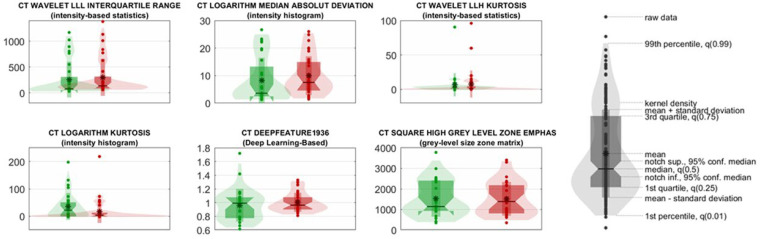
Violin plots of the six most significant radiomic features used in the classification model to discriminate between favorable (FP, red) and unfavorable prognosis (UP, green) in patients with metastatic melanoma. Each plot illustrates the distribution density, median, interquartile range, and individual data points for both classes. The selected features span different radiomic families, including intensity-based statistics, histogram-based features, texture metrics (GLSZM), and deep learning-derived attributes.

The external testing results (in which the prognosis of the single patient was associated with the one most frequently assigned to its metastatic lesions) showed that the classifier could predict subjects with a favorable prognosis with good accuracy (85%). A third of UP patients (35%) were correctly predicted (Figure 4).

Figure S1 shows the ROC curves of the other two models. The six most important radiomic features, used by the model to discriminate between the two classes, were as follows:

- CT wavelet LLL Interquartile Range and CT Logarithm Median Absolute Deviation reflect variability in lesion intensity, with higher values in UP patients indicating greater intratumoral heterogeneity;
- CT Wavelet LLH Kurtosis and CT Logarithm Kurtosis measure the peaks and tails of the intensity distribution, with elevated kurtosis values suggesting more irregular and aggressive lesion profiles in UP cases;
- CT DeepFeature1936, extracted from a ResNet50 convolutional neural network, captures abstract image patterns correlated with prognosis;
- CT Square High-Gray-Level Zone Emphasis (GLSZM) quantifies the prominence of large, high-intensity zones within lesions, with higher values in UP patients suggesting the presence of denser, potentially more aggressive tumor regions.

These features demonstrate statistically significant differences between prognostic groups (adjusted *p* < 0.05), supporting their role in model-driven stratification and underscoring the importance of radiomic heterogeneity in outcome prediction (Figure 4).

## 4. Discussion

New therapy approaches have improved stage IV MM patients’ survival. However, the overall mortality rate remains significant in MM patients due to disease complexity, and the prognosis remains highly variable [24,25]. Their management results are very complex because treatment failure is common, and many MM patients receive ineffective therapies associated with substantial side effects and undergo too many diagnostic exams at short time intervals [26,27]. Therefore, early patient stratification based on prognostic factors would enable clinicians to make more informed treatment decisions, reducing unnecessary and potentially harmful interventions. Precision medicine represents a promising approach in MM management, and radiomics could serve as a valuable tool for optimizing therapeutic strategies and patient monitoring. In recent years, several clinical studies have validated computational approaches in oncology [14,28]. A recent systematic review summarized the application of radiomics-based machine learning in predicting response to immunotherapy in melanoma across multiple studies from 2019 to 2022 [28]. In this context, our research group recently conducted a pilot study demonstrating that CT texture features at baseline and first post-treatment evaluation can serve as reliable predictors of OS and progression-free survival (PFS) in MM patients treated with new therapies [17]. In the present study, we estimated MM patient prognosis using a radiomics machine-learning model based on baseline metastatic lesion analysis. Radiomic analysis allows for the identification of lesion characteristics, leveraging AI to uncover complex interrelations [29]. Specifically, this method assesses pixel and voxel relationships, analyzing intensity variations within medical images and integrating them into classifiers for clinical classification and prognostic prediction [30]. Deep learning may further enhance this approach by extracting intricate and previously undetectable features, adding capabilities of conventional texture-based imaging techniques. Our analysis of pre-treatment CT images resulted in a model based on both conventional texture and deep features, with a receiver operating characteristic–area under the curve (ROC-AUC) of 82% in internal testing and 85% in external testing, aligning with the existing literature [30]. Variations in findings across studies may stem from differences in image acquisition protocols, patient demographics, or data pre-processing methods. Nonetheless, our results suggest that this texture alongside a deep learning-based approach holds strong predictive potential and could support clinical decision-making in MM prognosis assessment. In the external testing phase, the model could correctly classify the vast majority of FP patients, while one third of UP patients were identified as such. This result could have been initiated by the small cohort of analyzed patients in both the training and external testing phases. Although this study includes an external pre-test cohort, the data were collected from the same institution, limiting the generalizability of the results to different clinical settings or imaging technologies. We are currently designing a prospective multicenter study that will include a significantly larger and more heterogeneous patient population to overcome these limitations. The inclusion of multiple centers, different imaging protocols, and a broader range of clinical features will allow for a more comprehensive assessment of the generalizability and robustness of the model. Additionally, harmonized acquisition parameters and specific feature extraction pipelines will be adopted to minimize interinstitutional variability and ensure reproducibility. This strategy aims to validate the clinical applicability of our radiomics-based model in real-world settings. Various methods were used to reduce the risk of overfitting; however, we recognize that more extensive validation is needed. Our future work will focus on testing the model on independent multi-institutional datasets and heterogeneous imaging platforms. In addition, federated learning approaches could be considered to enable collaborative training of the model while preserving the privacy of patient data. These efforts aim to strengthen the reliability of the model and facilitate its clinical translation. Overfitting was mitigated using cross-validation and strict feature selection. Future evaluations on multicenter datasets will adopt nested validation and external testing to confirm robustness. It should be kept in mind that this work was intended as a preliminary study, and further investigations need to be carried out into a larger patient cohort. The most significant radiomic features show that a higher value of interquartile range, kurtosis median absolute deviation, and Square High-Gray-Level Zone Emphasis indicate high heterogeneity in tumor intensities and irregular tumor texture, and may correlate with a more aggressive disease. These features could provide valuable insights into tumor heterogeneity and intensity variations, aiding in prognosis estimation, treatment response prediction, and patient stratification. Their relevance was further verified through expert clinical review, confirming the consistency of these radiomic patterns with known prognostic indicators in metastatic melanoma. To improve the clinical interpretability of the model, we analyzed the most predictive radiomic features not only based on statistical significance, but also considering their clinical potential. Features such as interquartile range, kurtosis, and gray zone emphasis reflect intratumoral heterogeneity and intensity irregularities, which are known indicators of tumor aggressiveness. These descriptors, particularly when extracted from pre-treatment CT scans, could support patients’ early stratification and guide clinical decisions, such as treatment escalation or closer surveillance. Although no formal explainability techniques, SHapley Additive exPlanations (SHAP), or Local Interpretable Model Diagnostic Explanation (LIME) were applied at this preliminary stage, future work will incorporate these tools to better clarify model decision pathways and promote their clinical translation [31,32,33]. Moreover, radiomics-based machine learning classification has the potential to complement traditional diagnostic tools, further improving melanoma management. CT imaging was chosen for this study, as it accounts for approximately 75% of MM surveillance imaging, and its integration with machine learning may enhance patient outcome prediction [30]. However, several factors must be considered to mitigate bias and improve model generalizability, such as variations in CT acquisition protocols across different institutions [34]. Standardization of imaging protocols, including predefined CT parameters and cross-center image normalization, could reduce discrepancies and enhance reproducibility [35,36]. Furthermore, advanced AI techniques, such as federated learning, could enable robust model training without compromising patient privacy, thereby improving model generalization [37]. Frequent imaging follow-ups are essential for monitoring disease progression and treatment response in MM patients. However, in some cases, these follow-ups may be excessive and unnecessary for specific patient conditions [38]. Predictive models could help reduce the frequency of imaging examinations by identifying patients who actually require intensive monitoring while optimizing clinical resources and minimizing patient exposure to unnecessary procedures [39].

This study paves the way for future research on machine learning applications in MM management. It is important to recognize that machine learning algorithms may inherit biases from the training data, which can affect fairness and clinical applicability [28]. In this study, bias mitigation was partially addressed through class balancing and adherence to standardized radiomic workflows. However, further steps, such as external validation across diverse populations, the inclusion of clinical and socio demographic variables, and model interpretability analysis, are necessary to ensure ethical deployment and equitable outcomes. These aspects will be prioritized in future developments of the model.

However, some limitations should be considered. First, our model was developed using imaging data alone, without incorporating key clinical variables such as comorbidities or tumor molecular characteristics. Integrating clinical and molecular data could further enhance model performance and should be prioritized in future research. Additionally, longitudinal patient monitoring, tracking lesion evolution over time, could improve long-term outcome prediction. Moreover, larger patient cohorts and external validation across multiple institutions are necessary to confirm these preliminary findings. Our AI-based approach demonstrates strong predictive capabilities, indicating that radiomics and machine learning could become valuable assets in MM clinical management. The model’s robustness and high performance in survival prediction suggest that, with further optimization, this tool could support personalized treatment strategies and surveillance while optimizing healthcare resource allocation. Future evaluations will include additional performance metrics, such as precision, recall, and F1-score, to better capture model behavior in clinically unbalanced scenarios. Threshold selection will be optimized based on clinical priorities, balancing over- and under-treatment risks.

## 5. Conclusions

Radiomic analysis of CT images made it possible to extract quantitative features related to the texture, morphology, and intensity of MM lesions, providing a more comprehensive and accurate assessment of the risk of disease progression with respect to traditional methods. The results obtained with this approach show particularly favorable performance, with a significative value for sensitivity, specificity, and AUC, suggesting a possible integration of this technology into clinical practice. Radiomic integration in MM patient management could represent an important step toward more targeted and personalized medicine, improving therapeutic decisions and, consequently, patient quality of life. The adoption of predictive models based on advanced technologies such as machine learning could help overcome the challenges of the disease’s inherent variability and heterogeneity by providing an early and reliable prognostic assessment.

## Figures and Tables

**Table 1:** Principal characteristics of the study population.

	Internal Testing		Internal Testing	
	**FP**	**UP**	**FP**	**UP**
% Male	70.6%	60.0%	66.7%	50.0%
% Female	29.4%	40.0%	33.3%	50.0%
Median Number of Patients Lesion	1.88	2.8	2.0	2.6
Median Age	64.9	66.7	73.7	66.9
Median PFS (months)	33.8	2.2	44.3	6.0
Median OS (months)	56.7	20.8	96.7	32.5

FP (favorable prognosis), UP (unfavorable prognosis), PFS (progression-free survival), OS (overall survival).

**Table 2:** Performances of three radiomic-based models. Values are expressed as percentages with corresponding 95% confidence intervals (CI) in brackets. Internal testing results are reported both as mean and majority vote. * Statistically significant difference with *p* < 0.05. ** Statistically significant difference with *p* < 0.005.

	Metric	Training (%) [95% CI]	Validation (%) [95% CI]	Internal Testing (Mean) (%) [95% CI]	Internal Testing (Majority Vote) (%) [95% CI]
1	ROC-AUC	100 * [99–100]	80 ** [79–81]	82 ** [78–85]	82
	Accuracy	100 * [99–100]	74 ** [72–76]	75 ** [74–77]	77
	Sensitivity	100 * [99–100]	80 ** [78–82]	80 ** [78–83]	84
	Specificity	100 * [99–100]	67 ** [64–70]	70 ** [66–73]	68
	PPV	100 * [99–100]	77 ** [75–78]	75 ** [73–77]	75
	NPV	100 * [99–100]	77 ** [74–80]	76 ** [74–78]	79
2	ROC-AUC	67 ** [66–68]	56 ** [56–57]	49 ** [42–56]	47
	Accuracy	65 ** [64–66]	56 ** [54–59]	54 ** [62–83]	55
	Sensitivity	77 ** [76–78]	69 ** [63–75]	73 ** [62–83]	75
	Specificity	51 ** [48–54]	42 ** [37–48]	32 ** [28–37]	32
	PPV	64 ** [63–65]	59 ** [57–61]	55 ** [51–59]	56
	NPV	66 ** [65–67]	55 ** [49–61]	51 ** [41–61]	53
3	ROC-AUC	81 ** [80–82]	51 ** [48–54]	67 ** [65–69]	70
	Accuracy	74 ** [73–75]	52 ** [50–54]	60 ** [56–64]	67
	Sensitivity	79 ** [77–81]	61 ** [59–62]	76 ** [71–81]	81
	Specificity	68 ** [68–68]	42 ** [37–48]	43 ** [35–51]	50
	PPV	74 ** [74–75]	55 ** [49–60]	60 ** [57–64]	65
	NPV	74 ** [72–76]	48 ** [46–51]	61 ** [55–66]	70

## Data Availability

The data that support the findings of this study are available from the corresponding author upon reasonable request.

## Supplementary Materials

The following supporting information can be downloaded at: https://www.mdpi.com/article/10.3390/cancers17142304/s1, Figure S1: ROC Curve for the other two models obtained (from Internal Testing).

## Abbreviations

The following abbreviations are used in this manuscript:

ADASYN	Adaptive Synthetic Sampling
AI	Artificial Intelligence
AUC	Area Under the Curve
CT	Computed Tomography
CI	Confidence Interval
FP	Favorable Prognosis
FDR	Fisher Discriminant Ratio
GLCM	Gray-Level Co-occurrence Matrix
GLRLM	Gray-Level Run Length Matrix
GLSZM	Gray-Level Size Zone Matrix
IBSI	Image Biomarker Standardisation Initiative
ICIs	Immune Checkpoint Inhibitors
LIME	Local Interpretable Model Diagnostic Explanation
k-NN	k-Nearest Neighbors
MM	Metastatic Melanoma
NGTDM	Neighborhood Gray Tone Difference Matrix
NGLDM	Neighboring Gray Level Dependence Matrix
NPV	Negative Predictive Value
OS	Overall Survival
PCA	Principal Component Analysis
PFS	Progression-Free Survival
PPV	Positive Predictive Value
ROC	Receiver Operating Characteristic
ROC-AUC	Receiver Operating Characteristic-Area Under the Curve
SHAP	SHapley Additive exPlanations
SVM	Support Vector Machine
UP	Unfavorable Prognosis
VOI	Volume of Interest
