# Supplementary material for: Metastatic Melanoma Prognosis Prediction Using a TC Radiomic-Based Machine Learning Model: A Preliminary Study
Source: Cancers (Basel). 2025 Jul 10;17(14):2304. doi: 10.3390/cancers17142304 (PMC12293981; doi:10.3390/cancers17142304)
Supplement: Supplementary file 1 [file cancers-17-02304-s001.zip › cancers-3722477-supplementary.pdf]

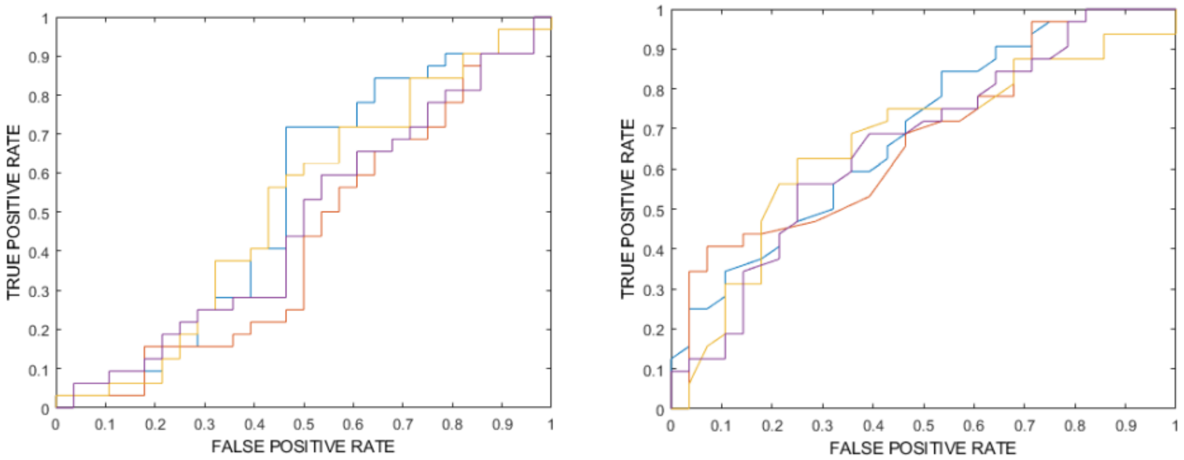

Figure S1. ROC Curve for the other two models obtained (from Internal Testing). Relationship between the True Positive Rate (y-axis) and the False Positive Rate (x-axis) for different threshold values.
